# Impact of *Moringa oleifera* Leaves on Nutrient Utilization, Enteric Methane Emissions, and Performance of Goat Kids

**DOI:** 10.3390/ani13010097

**Published:** 2022-12-27

**Authors:** Vandana Kumari Leitanthem, Parul Chaudhary, Sanjit Maiti, Madhu Mohini, Goutam Mondal

**Affiliations:** Animal Nutrition Division, ICAR-National Dairy Research Institute, Karnal 132001, Haryana, India; Dairy Extension Division, ICAR-National Dairy Research Institute, Karnal 132001, Haryana, India

**Keywords:** enteric methane emissions, *Moringa oleifera*, goat, growth performance

## Abstract

**Simple Summary:**

This study concludes that supplementation with *Moringa oleifera* leaves in concentrated mixtures improves nutrient digestibility, growth performance, immunity status, and antioxidant activity in goat kids under different feeding regimens. Supplementation of *Moringa* leaves—which are rich in protein, minerals, and beneficial biomolecules—can be used up to the level of 10–20% in concentrated mixtures for better performance in goats. These also meet the essential necessities of small ruminants and overcome the problem of enteric methane emissions. Hence, they may be recommended for goats as a protein source supplement and to mitigate the methane emissions from ruminants.

**Abstract:**

The development of different innovative feed resources for livestock is important to provide the essential nutrients and diminish the emission of greenhouse gases. The purpose of the present experiment was to study the response of replacing concentrate with *Moringa oleifera* leaves in terms of the nutrient intake, digestibility, enteric methane emissions, and performance of goat kids with a berseem-fodder-based diet under different roughage (R)-to-concentrate (C) ratios. Twenty-four goat kids (3 months of age) were distributed into four groups of six animals each, using a randomized block design (RBD). Kids of Group I (control) were fed a basal diet with 70R:30C without any tree leaf supplementation. Group II kids were fed with 60R:40C, where 10% of the concentrate mix was replaced with *Moringa* leaf (ML powder). In Group III, kids were fed with 70R:30C with 20% ML replacement. In Group IV, kids were fed with 80R:20C with 20% ML replacement. A metabolic trial was conducted after 180 days of feeding to assess the impact of ML on blood metabolites, antioxidant status, immunity parameters, and enteric methane emissions. The results revealed that dry matter digestibility, organic matter, and NDF were better (*p* < 0.05) in ML-treated kids (GII and GIII) compared to GI. Feed conversion and average daily gain were also enhanced (*p* < 0.05) in the treated groups as compared to controls. Total blood protein and albumin were increased in GII and GIII kids compared to GI. Plasma cholesterol levels were decreased (*p* < 0.001) in GII, GIII, and GIV as compared to GI. Glutathione peroxidase, catalase, and superoxide dismutase enzyme activities were also enhanced in GII, GIII, and GIV compared to controls. ML supplementation improved cell-mediated immunity and humoral immunity responses in goat kids. Enteric methane emissions decreased in the treated groups as compared to the controls. *Moringa oleifera* leaf may be used up to the level of 10–20% in concentrate mixes to improve digestibility, blood biochemical parameters, immunity status, and antioxidant activity in goat kids. Supplementation of ML not only enhanced the digestion and health of goat kids, but also decreased their methane emissions.

## 1. Introduction

Goats make up about 27.74% of the total livestock population in the Indian subcontinent, followed by cattle (35.92%), and play an important role in sustainable animal agriculture. Almost 35 million farmers are directly involved in goat rearing. Goat rearing is generally practiced under semi-intensive rearing systems, and most farmers use approximately 20–30% concentrated mixtures for their goats [1]. Recently, a challenge faced by animal producers is the shortage and high price of concentrated feeds. Poor quality of animal feed is a major factor in decreasing animal productivity in tropical countries [2]. Due to the constant increase in competition between humans and livestock for food, along with the limited land area for the cultivation of fodder and food grains, the prices of protein sources are continuously rising [3]. A protein-deficient diet causes inadequate growth and reduced production performance. Therefore, the search for alternative protein sources for livestock is of immense importance.

Recently, many studies have shown that tree leaves can be a good source of crude protein and can positively influence the environment by mitigating methane emissions [4,5]. However, many factors may affect the utilization of such byproducts in ruminants’ nutrition, such as the protein and fiber contents or the concentrations of bioactive components, which can directly influence rumen fermentation parameters [6]. *Moringa oleifera* (ML) is one of the potential tree fodders, also known as the miracle tree, and is a native tree in India [7]. Its leaves have nutritional value and are rich in crude protein content (29.40%), minerals such as calcium (2.65%) and phosphorus (0.304 g/100 g), and vitamin C (188–279 mg/100 g) [8]. Additionally, approximately 47% of the protein in *M. oleifera* leaves is rumen bypass protein with a good amino acid profile. ML meal contains nine times more protein than yogurt, with a good feeding effect, and can be used as a protein substitute in animal feed. Consumption of ML as an animal feed supplement in tropical countries has numerous benefits compared to other plants, as it can tolerate chill and drought stress, can be simply altered and assimilated by the animals, and has antinutritional properties [9]. The presence of alkaloids, polyphenols, and polysaccharides in ML makes it useful as a feed additive [10]. Nouman et al. [11] described the usage of ML for animal feed in ruminants, leading to improvements in microbial protein synthesis in the rumen. Numerous studies have been conducted that provided information on the effects of ML on the performance and quality of milk in goats and cows [12]. Al-Juhaimi et al. [13] reported that ML improved the immune system and oxidative status in goats through bioactive composites. Supplementation with ML improved the ruminal fermentation, intake of feed, and milk quality in Nubian goats [14].

Total anthropogenic greenhouse gas (GHG) emissions in terms of CO_2_ equivalents represent all economic sectors. Livestock contributes approximately 14.5%, and the overall agricultural sector—including the livestock—emits roughly 23.20% of emissions in India. Goats contribute approximately 5.38% of enteric methane emissions within agriculture. Methane emissions not only contaminate the atmosphere, but also cause energy losses (2–12%) and low production efficacy in animals. Animal feed is one of the most important factors that influence the enteric methane emissions and their efficiency. Dietary interventions have been found to be efficient in ruminants to reduce the enteric methane production [15]. Nutritional methods and numerous native feeds/tree leaves may reduce enteric methane release from cattle production by up to 15% [16]. An effective fed may also produce less compost N and, consequently, less anaerobic fermentation to discharge methane, ammonia, and nitrous oxide into the air. The usage of various plants with anti-methanogenic potential has been proposed as a way to minimize methane emissions through the presence of secondary metabolites. *Leucaena leucocephala* legumes have shown a good methane mitigation effect when constituting up to 30–35% of diet dry matter in cattle [17]. ML is a natural feed and cost-effective protein source that can modify the fermentation pathways, inhibit the growth of methanogens, and alleviate methane from buffaloes [18]. ML is a low-priced supplement as compared to other protein supplements such as soybeans and sesame [19]. ML is easily adapted and digested by animals, and also contains a variety of compounds that show the antimicrobial properties and improve the feed utilization and performance of ruminants [20]. Supplementation of ML in goat food improved the fat levels in milk and decreased the abundance of *Methanobrevibacter ruminantium*, which is involved in methane production [21]. Application of ML (4%) in the diets of mice improved their size, weight, and survival rates, as reported by Zeng et al. [22]. Addition of 3.5% ML to the diet improved antioxidant status, milk production, and reproductive functions in goats [23]. However, a comprehensive study replacing concentrate mixture has not been conducted so far. We hypothesized that ML with high CP and bioactive molecules could regulate ruminal fermentation and be utilized in ruminants. Only a few studies on methane emissions and animal performance have been carried out on male goat kids. Therefore, the goal of the present study was to observe the effects of *Moringa oleifera* leaves on the methane emissions and production performance of male goat kids under organized farm conditions.

## 2. Materials and Methods

### 2.1. Farm Description and Feed Preparation

The present study was conducted in the Livestock Research Centre, ICAR-NDRI, Karnal, Haryana, India. This institution is situated at 29°42′ N and 79°54′ E, 834 feet above sea level. The maximum and minimum temperatures during summer are 45 °C and 4 °C, respectively, with a diurnal variation of 15–20 °C. Research practice and maintenance were as per the standard of the Institute Animal Ethics Committee, and consent was also obtained from the committee, with IAEC approval no. 92/16. This research was conducted from December 2017 to July 2018. *Moringa oleifera* leaves were collected from the NDRI farm, shade- and oven-dried, and ground to a powder before being packed in airtight polythene bags, while *Trifolium alexandrinum* (berseem var. BL42) and the concentrate mixtures were oven-dried at 60 °C. Desiccated samples were crushed and sieved (1 mm) using an electrically operated Wiley mill. After complete drying, the samples were ground and placed in sample bottles for further use. 

Secondary metabolites such as tannins, saponins, total phenols, and flavonoids were estimated using the standard protocols. Tannin estimation was carried out using the method described by Nwinuka et al. [24]. Tannic acid (1 mg/mL) was used as the reference. The plant leaf extract (1 mL) was mixed with Folin–Ciocalteu reagent (0.5 mL) and sodium carbonate solution (1 mL). The total volume was made up to 5 mL. Tannin concentrations were determined by measuring absorbance at 755 nm and calculated as tannic acid equivalents a from standard curve.

To estimate the total saponins, methanolic extract of leaves (500 µL) and anisaldehyde reagent (500 µL, 0.5%) were mixed in a test tube and left for 10 min. Sulfuric acid (5%, 2 mL) was added to tubes, mixed properly, and kept in a water bath at 60 °C for 10 min. The tubes were cooled, and the absorbance was measured at 435 nm [25].

Total phenolic content (TPC) in the methanolic extract of ML was determined using the Folin–Ciocalteu reagent assay. Folin–Ciocalteu reagent (750 mL), sodium carbonate (7.5%, 2 mL), and methanolic leaf extract (200 mL) were added to a tube. The mixture was diluted with deionized water to 7 mL, and then left at room temperature in the dark for 2 h. The absorbance was measured at 765 nm using a spectrophotometer and calculated using gallic acid equivalents (g/110 g of extract) [26].

Briefly, methanolic extract (1 mL) was added to a 10 mL volumetric flask containing water (4 mL) for total flavonoid estimation. Sodium nitrate (0.3 mL, 5%) was added to the flask, followed by aluminum chloride (0.3 mL, 10%) at 5 min and sodium hydroxide (2 mL, 1 M) at 6 min. Then, 2.4 mL of water was added to the flask, and absorbance was measured at 510 nm and calculated as epicatechin equivalents (mg/g) [27].

### 2.2. Experimental Design

Twenty-four apparently healthy kids (3–4 months of age) were kept independently in experimental sheds and sustained on similar basal feed (roughage and concentrate). Proper deworming was performed on all animals during the initial period of the experiment itself. Prior to the experimental feeding, feed intake was recorded for 10 days. During this period, the animals also adapted to the changed environment. The body weight of the animals was initially recorded for 2 consecutive days, and thereafter at 15 day intervals, with the animals separated into 4 groups, each consisting of 6 animals, in randomized block design. Group I was the control group, with a 70R:30C ratio (roughage to concentrate) made up of berseem and concentrate but no ML supplement. Group II was fed with 60R:40C containing 36% concentrate and 4% *Moringa oleifera* leaf powder. In Group III, the goats were fed with 70R:30C containing 24% concentrate and 6% *Moringa oleifera* (Table 1). In Group IV, the goats were fed with 80R:20C containing 16% concentrate and 4% ML. All four groups’ diets were isonitrogenous in nature.

### 2.3. Metabolic Trial

The metabolic trial was conducted at the end of experimental period for a 7-day collection period to assess the nutrients’ digestibility and nitrogen balance in a specially designed cage. Animals were kept in this cage for one week prior to the metabolic trial to allow them to adjust to their surroundings. The height of the gate of the cage was 63 cm, of which the free height was 30 cm and the iron plate height was 33 cm. The height of the cage from the ground to the upper end and the cage floor was 200 and 30 cm, respectively, with a width of 263 cm. Data on feed offered, feed refusal, and feces and urine voided by the individual animals in all treatment groups were recorded on daily basis. Urine samples were collected in plastic bottles from the metabolic chamber and preserved for further analysis.

#### 2.3.1. Analysis of the Mineral Nutrients Composition of the Feed

Dried (0.5 g) and crushed ML feed samples were weighed into digestion tubes, and tri-acid mixture (10 mL) was added for digestion in a Kelplus micro digestion assembly. The absence of white fumes and black particles in the residues suggested that the samples had been completely digested. The digested samples were then filtered using filter paper. The filter paper was rinsed many times with double-distilled water and an inductively coupled plasma (ICP) optical emission spectrometer (iCap 6000, Thermo Scientific, Cambridge, UK) was used for the analysis of minerals such as calcium, magnesium, iron, copper, and zinc, while phosphorus (P) was estimated using spectrophotometric (Thermo Fisher Scientific) methods [28].

#### 2.3.2. Analysis of Samples for Chemical Composition

Different samples of feed and feces were collected during the metabolic trial on a daily basis and dried at 60 °C in an oven to estimate the dry matter. Total N content was estimated using the Kjeldahl method. The CP content for each feed sample was estimated by multiplying the content of nitrogen by 6.25. Ether extract (EE) content was estimated using a solvent extraction process [29]. Total ash content was estimated via combustion in a muffle furnace. Neutral and acid detergent fiber (NDF, ADF) and lignin contents were assessed using the method described by Van Soest et al. [30].

#### 2.3.3. Growth Performance

The animals were weighed before being fed and given water in the morning on two consecutive days at the start of the experimental feeding, and then at two-week intervals for the duration of the six-month trial. An average of two days was used to calculate body weight. The increase in body weight at weekly intervals was used to calculate the growth rate, and the ratio of feed intake to gain was used to assess feed conversion efficiency (FCE). FCE was calculated by the ratio of the ADG of an individual animal to its daily DMI. The average daily gain (ADG) was determined by dividing the difference between the initial and final live weight by the number of days. Gross energy (GE) values of roughage, concentrates (including ingredients), and basal diets were calculated based using the following equation: GE (Kcal/100 g DM) = (2.62 × % CP) + (8.37 × % EE) + (4.2 × % CF), and DE and ME were calculated directly from GE.

#### 2.3.4. Methane Estimation

The enteric methane (CH_4_) production was estimated using the SF_6_ tracer gas technique for 5 days [31]. In this method, the permeation tubes were prepared by filling them with specific amounts of SF_6_ and inserted into the rumen with a known release rate. Each animal was fitted with a halter and a capillary tube attached to an evacuated sampling canister set to fill halfway in 24 h. Samples from the animal’s mouth and nose were taken as the vacuum in the sampling canister gradually dissipated. Background CH_4_ and SF_6_ concentrations were measured for each day by placing one sampling kit in a naturally ventilated house. The amounts of CH_4_ and SF_6_ in the animal samples were then corrected for background concentration [32]. Following sample collection, the canister was pressurized with nitrogen, and the concentration of SF_6_ was determined using a gas chromatograph (Nucon 5700, Nucon Engineers, New Delhi, India) equipped with an electron capture detector (250 °C) and a 3.3 m molecular sieve column. To estimate the CH_4_ concentration, another gas chromatograph was equipped with flame ionization detector (100 °C) and a stainless-steel column packed with Porapak-Q. In both gas chromatographs, the injector and column were set to 50 and 40 °C, respectively. 

#### 2.3.5. Collection of Blood Samples

Blood samples (8 mL) were collected from six goat kids in each group on days 0, 30, 60, 90, 120, 150 and 180 in the early morning before the provision of feed and water. The samples were taken from animals into vacutainers containing heparin. Immediately, the vials were slightly rolled between the palms for proper mixing, kept in ice box, and stored in the laboratory for further analysis. Blood biochemical analysis for glucose, protein, albumin, and globulin contents was carried out using kits purchased from Recombigen Lab. Pvt. Ltd., New Delhi, India (Cat.no. GLU-L1001, TTP-L250, ALB-L-100). The activities of enzymes such as glutathione peroxidase (GPx), catalase (CAT), and superoxide dismutase (SOD) were estimated using the methods described by Paglia and Valentine [33], Aebi [34], and Madesh and Balasubramanian [35].

### 2.4. Immunological Analysis

#### 2.4.1. Cell-Mediated Immune Response (CMI) 

All of the goats were injected intradermally with phytohemagglutinin-P (150 µg PHA-P) in the neck to measure the cell-mediated immune response in terms of delayed-type hypersensitivity (DTH) [36]. The DTH response was measured using a Vernier caliper and expressed as the percentage increase in skin thickness. 

#### 2.4.2. Humoral Immune Response (HI)

HI was evaluated by hemagglutination tests using sheep red blood cells (SRBCs). Animals were injected with SRBCs (2 mL, 10%) on the 60th day of the experiment, while the booster dose was administered 7 days later. Blood was collected on days 0, 7, 14, 21, and 29 (before injection) to estimate the antibody response. Approximately 2 mL of blood was collected in sterile serum collection vacutainers, and the collected sera were transferred aseptically to washed, marked plastic vials and stored at −20 °C until further analysis by the hemagglutination (HA) test. Antibody titers were expressed in a log_2_ basis [37].

### 2.5. Statistical Analysis

The results were statistically examined by means of SPSS software using the general linear model procedure through one-way and two-way analysis of variance for growth parameters, and repeated-measures ANOVA at *p* < 0.05 with Tukey’s test for blood parameters. The values of the above parameters are presented as the mean ± standard error.

## 3. Results

### 3.1. Mineral and Chemical Composition of Berseem Fodder, Concentrate, and Moringa oleifera Leaves

The calcium content was highest in berseem (1.92%), followed by *Moringa oleifera* (1.85%) and the concentrate mixture (1.37%), while the phosphorus content was highest in the concentrate mixture (0.79%), followed by berseem fodder (0.38%) and *Moringa oleifera* (0.15%). The magnesium content was highest in *Moringa oleifera* (4.81%), followed by the concentrate mixture (0.81%) and berseem fodder (0.40%). The Fe content was 287 ppm in the concentrate mixture, 289 ppm in the berseem fodder, and 330 ppm in the *Moringa oleifera*. The levels of Cu and Zn (ppm) in the concentrate mixture (29.21 and 49.00, respectively), berseem fodder (4.61 and 16.41, respectively), and *Moringa oleifera* (9.21 and 26.72, respectively) are presented in the Supplementary Materials (Table S1). The chemical composition of the different feeds is presented in Table S2. The OM, CP, and EE contents (%) were higher in ML (89.62, 23.37, and 7.13%, respectively) in comparison to the concentrate feed (89.26, 20.14, and 4.45%, respectively) and berseem fodder (87.22, 17.56, and 2.98%, respectively). On the other hand, the NDF, ADF, and TA contents were lower in ML (26.86, 18.70, 10.03%) compared to berseem (59.29, 39.33, and 12.78%, respectively). On the basis of dry matter percentage, the value of each phytochemical component—i.e., tannin, saponin, TPC, and TFC—in *Moringa oleifera* leaves was calculated. The TPC content was the highest (4.28%), followed by TFC (3.61%), tannin (2.02%), and saponin (1.01%).

### 3.2. Chemical Composition of Feed

The chemical composition of the total mixed ration (TMR) fed to the different groups was isonitrogenous (CP concentration = 18%), and the remaining composition was broadly similar, with the exception of NDF and ADF contents (Table 2). The TMR values of DM, OM, CP, and EE in GII were the highest, as 48.17, 87.98, 18.72, and 3.49%, respectively, followed by GIII (40.17, 87.77, 18.53, and 3.39%, respectively), GI (43.89, 87.75, 18.33, and 3.33%, respectively), and GIV (34.65, 87.55, 18.21, and 3.25%, respectively). The contents of NDF, ADF, hemicellulose, cellulose, and TA were lower in GII (45.14, 28.66, 16.48, 18.11, and 12.01%, respectively) as compared to GI (48.58, 31.16, 17.42, 19.44, and 12.25%, respectively), GIII (48.77, 31.58, 17.20, 20.22, and 12.21%, respectively), and GIV (52.28, 34.22, 18.06, 21.81, and 12.44%, respectively).

### 3.3. Feed Intake and Digestibility of Nutrients in Different Groups

The digestibility coefficients of DM and OM were highest (*p* = 0.02) in GII (69.70% and 71.89%, respectively) and GIII (70.82% and 71.76%, respectively), followed by GIV (67.68% and 69.75%, respectively), while the lowest coefficients were observed in GI (66.84% and 69.56%, respectively). The digestibility of crude protein, EE, and ADF (%) was similar in all of the groups. NDF digestibility was higher (*p* < 0.05) in GIII (56.04%) and GII (54.91%) as compared to GIV (53.35%) and GI (52.94%) (Table 3). Organic matter intake was similar in all of the groups (*p* < 0.07), while DMI was higher (*p* < 0.01) in GII and GIII as compared to GI and GIV. Similarly, CP and NDF intakes were also higher in GII and GIII as compared to GI and GIV. Overall TDN intake was higher in GII and GIII as compared to GI and GIV.

### 3.4. Overall Performance of Goats after 180 Days of the Feeding Trial

The initial body weight of goat kids in GI, GII, GIII, and GIV was similar in all groups (Table 4). The final weight in GII (32.50 kg) and GIII (31.84 kg) was greater (*p* < 0.01) than that in GI and GIV (28.43 and 29.15 kg, respectively). Overall weight gain was also higher (*p* < 0.004) in GII (18.18 kg) and GIII (17.48 kg) than GI (14.13 kg) and GIV (14.59 kg). ADG was highest (*p* < 0.001) in GII (101.01 g), followed by GIII (95.68 g), GIV (81.42 g), and GI (79.22 g). However, FCR (gain: intake) was similar (*p*-value) in all of the groups.

### 3.5. Impact of ML on N Balance in Goats

The N intake was highest (*p* < 0.01) in GII (19.87), followed by GIII (19.13), GIV (17.67), and GI (17.75 g/d). The fecal and urinary N contents were similar in all of the groups (Table 5). Total N loss was also highest in GII and GIII (17.01 and 16.53 g/d, respectively), followed by GI and GIV (15.46 and 15.23 g/d, respectively). The N balance was highest (*p* = 0.02) in GII (2.87) and GIII (2.60), followed by GIV (2.44), while the lowest was found in GI (2.29 g/d). However, absorbed N (%) was also similar in all of the groups. The percentage of nitrogen intake retained and absorbed was statistically similar. 

### 3.6. Enteric Methane Production in Goats

Methane emission expression (g/d, MJ/d, g/kg DMI) was not varied between the groups; however, the trend (*p*-values 0.07 to 0.12) indicated that there was a close and inverse association between enteric methane emission and the presence of ML in the diet (Table 6). 

### 3.7. Effects of ML on Blood Parameters and Antioxidant Activities in Goats

Glucose concentrations were similar (*p* = 0.72) in all groups (GI, GII, GIII, and GIV), at around 64 mg/dL (Table 7). The total blood protein contents in GII and GIII (6.97 and 6.99 g/dL, respectively) were higher (*p* < 0.001) than those in GIV and GI (6.77 and 6.74 g/dL, respectively). The same trend was observed in albumin content; the blood albumin levels in GII and GIII (3.66 g/dL) were significantly different from those in GIV and GI (3.44 and 3.38 g/dL, respectively). However, similar globulin levels (3.36, 3.31, 3.32, and 3.34 g/dL) were observed in GI, GII, GIII, and GIV, respectively. Similarly, the A:G ratio was also similar in all of the groups (*p* = 0.005). Blood plasma cholesterol levels were highest in GI (97.36 g/dL) (*p* < 0.001), followed by GIV (93.89 g/dL), GII (93.70 g/dL) and, finally, GIII (92.36 g/dL). The levels of blood plasma AST, ALT, and ALP were found to be within the biological range, and no differences were observed between the groups. Antioxidant enzyme activities—i.e., GPx, CAT, and SOD—were highest (*p* < 0.001) in GIII (15.03, 55.03, and 93.40, respectively), followed by GII (14.80, 54.80, and 93.28, respectively) and GIV (14.68, 54.68, and 93.04, respectively), while the least activity for GPx, CAT, and SOD was observed in the control group (GI).

### 3.8. Effects of ML on Cell-Mediated (CMI) and Humoral Immune (HI) Responses in Goats

**Figure 1:**
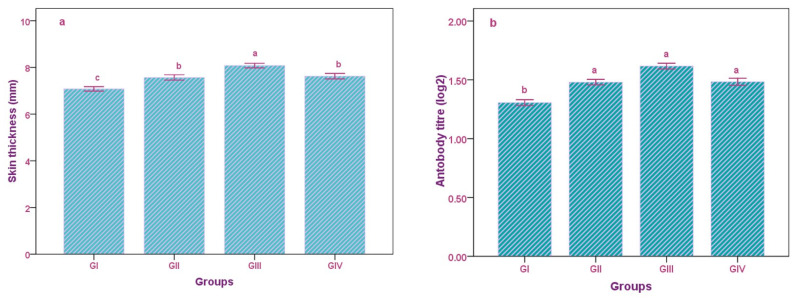
Impact of replacement of concentrate with *Moringa oleifera* leaves in different groups of goat kids on (**a**) cell-mediated immunity and (**b**) humoral immunity. Bars followed by different letters indicate that the mean ± SEM values showed significant differences (*p* < 0.05).

CMI was checked in relation to DTH response towards PHA-P, and all kids displayed a positive response (Figure 1a; Table S3). The skin thickness in GII (8.08 mm) was the highest (*p* < 0.001), followed by GIV (7.62 mm), GII (7.57 mm) and, finally, GI (7.08 mm). The humoral immunity (HI) was assessed as the antibody response to sheep erythrocytes (SRBC) using the HA test. The results revealed that the titers reached the maximum value on the 14th day post-inoculation, showing a declining trend thereafter up to the 21st day post-inoculation (Figure 1b; Table S4). The antibody titer against SRBC was highest in GIII (1.61 log_2_), followed by GII, GIV (1.48 log_2_), and GI (1.30 log_2_).

## 4. Discussion

Due to the poor quality of animal feeds, animal production is often restricted and contributes to higher GHG emissions. In many countries, feedstuffs—especially protein sources that are necessary for animals’ growth and development—have become exceedingly expensive. Therefore, it is necessary to search for a replacement source of feed that is palatable, abundant in proteins and minerals, and affordable for ruminants, as well as reducing methane emissions. ML serves as a healthy, cheap, and reasonable source of nutrients, minerals, and proteins for ruminants. One of the best ways to improve ruminants nutritional condition is by supplementation with plant-derived feed with adequate nutritional value [38]. *Moringa oleifera* leaves are suitable for animal feed because they are high in essential nutrients and low in antinutritional factors. More research has been carried out on the impacts of ML in fattening goat kids; however, feeding ML to the kids may reduce GHG emissions from the livestock sector. Therefore, this study investigated the feeding of male goat kids with *Moringa oleifera* leaves (4%, 6%, and 4%) as a protein source with different roughage-to-concentrate ratios (60R:40C, 70R:30C, and 80R:20C) to reduce the enteric methane emissions.

We observed that ML had higher magnesium and iron contents than berseem and the concentrate mixture. Trace minerals such as zinc, iron, copper, selenium, and manganese are essential for various biochemical reactions, and play important roles in tissue repair, protein metabolism, and boosting the immune system [39]. 

*Moringa oleifera* is an innovative fodder that contains essential nutrients required for livestock, enhanced feed utilization efficiency, and animal performance. In this study, it was observed that the CP content was higher in ML as compared to berseem and the concentrate mixture, while the NDF content was lowest in ML, suggesting that ML is palatable compared to other feeds [10]. Kholif et al. [40] observed that supplementation with ML extract in Nubian goats improved the digestibility of OM, dry matter, and NDF, consistent with our results. This may be because the presence of phenolics, tannins, and saponins present in ML alters the rumen environment positively for utilization of energy by rumen microbes [41]. On the other hand, ML feed supplementation (100%) significantly enhanced the CP and NDF in Bengal goats, as reported by Sultana et al. [42]. 

This study also revealed that the digestibility of selected nutrients was enhanced in GII (60R:40C), where 10% of the concentrate was replaced with ML, and in GIII (70R:30C), with 20% replacement of the concentrate with ML as compared to the control group. This may be because the presence of secondary metabolites such as tannins, saponins, and flavonoids in ML may act as a hydrogen sink and thereby help in reducing methane production. Improvement in microbial fermentation was observed when animals were fed with ML, which improved their nutrient consumption along with roughage [43]. The presence of phenolic composites and secondary metabolites in ML augmented the digestibility rate and sustained an excellent ruminal atmosphere with a good feeding value [44]. However, feed conversion efficiency remained statistically similar, indicating that ML may also have enhanced digestibility and excretion.

The goats fed with ML had a higher daily weight gain, and the highest was found in GII, GIII, and GIV as compared to the control group. This may have been due to the augmentation in protein consumption present in *Moringa* leaves, which improved the feed intake and digestibility and, thus, the growth, due to the presence of some bioactive metabolites. Feeding the sheep with concentrate along with ML (25%) significantly enhanced the intake of CP, weight gain, and nutrient digestibility due to the presence of some unknown biomolecules [45,46]. Hassan et al. [47] found that supplementation with ML improved the feed intake rate, body weight gain, and feed conversion efficiency—even in broiler chicks under heat stress. Aregheore [48] observed the positive impact of ML (20 and 50%) feed on the weight and digestibility of DM, CP, and OM in goats.

Nitrogen dynamics and availability were altered in this study, shown to be higher in GII and GIII as compared to the control group, and essential for adequate bacterial protein synthesis [49]. Utilization/absorption of ammonia-N in GII and GIV was the highest (20.12 and 19.15%, respectively), while the lowest was in GI (18.81%). ML is known as a better source of amino acids, improving the consumption of dietary N and increasing the production of dairy animals [50]. Meanwhile, Kholif et al. [51] reported that the application of ML decreased ruminal ammonia-N concentrations due to the presence of tannins and phenols, which reduce the degradability of rumen proteins. 

Methane emissions in GII (60R:40C) with 10% of concentrate replaced with ML and GIII (70R:30C) with 20% replacement of concentrate with ML were decreased as compared to the control group. This might have been due to the presence of phenols, tannins, and saponins in ML. The presence of α-linolenic acid, tannins, and phenolics had antimicrobial effects, which can be a main cause of methane reduction [52,53]. Supplementation of ML to replace soybean meal reduces methane production and ammonia-N in steers and goats. Incorporation of ML along with yeast culture in goats reduced methane emissions to the clean environment, as reported by Pedraza-Hernandez et al. [54]. *Paulownia* leaves are high in bioactive components and help to lower the rumen methanogen count, resulting in decreasing methanogenesis rates [5,6]. 

Blood parameters are well-known indicators that can provide information about animals’ feeding and health status. Feed quality may cause alterations in the blood metabolites [55]. Any change in glucose levels indicates that the animal is in stress or under a lot of pressure for production. The presence of iron in ML is known to improve Hb levels for better animal health. Total protein and blood albumin levels were also higher in the treated groups (GII and GIII) compared to the controls. Cholesterol levels were found to decrease in treated kids as compared to the control group. This may be due to the supplementation of ML, which contains phenols—especially saponin—resulting in reduced levels of cholesterol. The levels of serum cholesterol and triglycerides were decreased in animals fed with ML [56,57]. 

It is known that liver enzymes such as aspartate aminotransferase (AST) and alanine aminotransferase (ALT) are indicators of hepatic health, and increased presence of these enzymes in the blood indicates inflammation and/or metabolic disorders [58]. The decreases in AST and ALT levels in GII and GIII indicate that ML did not have any undesirable effects on the liver. ML supplementation also exerted a protective effect on the liver, enhancing the immune response and intestinal health of broilers [59]. Levels of antioxidant enzymes such as GPx, CAT, and SOD were higher in ML-treated kids as compared to the control group. ML fed to sows and piglets enhanced the serum total protein, GSH-Px, SOD, and CAT contents, as reported by Sun et al. [60]. ML contains phenols and has antioxidant properties that prevent oxidative injury in animals by increasing their antioxidant enzyme activities [61]. Moyo et al. [62] also observed significant elevation in CAT and SOD activities in goats, along with a reduction in MDA levels, when supplementing the feed with ML. Supplementation of feed with ML (1.6 and 3.2%) in Beetal goats’ feed increased plasma protein content, along with CAT, POD, and SOD activities [63].

The DTH test is used to assess the response of the skin to intradermal inoculation with antigens, which depends on antigen-specific memory T-cells. T-cell activation causes the release of lymphokines, which are involved in the accumulation of macrophages, increase vascular permeability, and cause vasodilatation and inflammation. They also activate the phagocytic activity and lytic enzymes for more effective killing of microbes. The results of our experiments revealed that ML can be used to improve the immune systems of ruminants. This may be due to the ML, which contains the vitamins C, A, and K, and stimulates the immune system by increasing T-cell proliferation, cytokine production, and immunoglobulin synthesis, which are involved in the inflammatory response and cause increased skin thickness [59]. The presence of amino acids in ML is also responsible for the formation of immunoglobulins and major histocompatibility complexes, which mediate the DTH reaction, although ruminal degradation and further absorption in the system have not been studied thoroughly, and need further validation. 

Humoral immunity includes the interaction of B cells with antigens and their proliferation into plasma cells, causing the secretion of antibodies. Antigens bind to antibodies, which act as effectors in the humoral response, neutralizing the former by crosslinking to form clusters that are ingested by phagocytic cells. Immunoglobulins, which are the products of amino acids and glycoproteins, are essential to the immune system. ML contains copper, which is involved in the functioning of ceruloplasmin in the immune response. The presence of zinc, vitamins, and selenium is also vital for the development of B lymphocytes [64].

## 5. Conclusions

This study concluded that *Moringa oleifera* leaves are a good source of nutrients (e.g., protein, trace minerals), and that the replacement of concentrate with *Moringa oleifera* up to the level of 10–20% can improve nutrient digestibility, growth performance, immunity status, and antioxidant activity in goats fed diets with different roughage-to-concentrate ratios prevalent under field conditions. Hence, it may be recommended to use such feed for better performance in goats as a supplementary protein source. Further study of different animals with different concentrations of ML will be needed, which might help to improve our understanding of microbial metabolic functions in the rumen under in vitro and in vivo conditions.

## Figures and Tables

**Table 1:** Different groups and feed ratios used in the experiment.

S.No.	Groups	Roughage (Berseem %)	Concentrate (%)	*Moringa oleifera* (%)
1.	Group I	70	30	0
2.	Group II	60	36	4
3.	Group III	70	24	6
4.	Group IV	80	16	4

**Table 2:** Chemical composition (%) of TMR in different groups of goats: GI (control), GII (60R:40C with 10% of concentrate replaced with ML), GIII (70R:30C and 20% replacement of concentrate with ML), and GIV (80R:20C and 20% replacement of concentrate with ML).

Attributes (%)	GI (70R:30C)	GII (60R:40C)	GIII (70R:30C)	GIV (80R:20C)
Dry matter	43.89 ± 0.20	48.17 ± 0.12	40.17 ± 0.17	34.65 ± 0.14
Organic matter	87.75 ± 0.15	87.98 ± 0.19	87.77 ± 0.18	87.55 ± 0.16
Crude protein	18.33 ± 0.07	18.72 ± 0.14	18.53 ± 0.13	18.21 ± 0.08
Ether extract	3.33 ± 0.04	3.49 ± 0.10	3.39 ± 0.05	3.25 ± 0.06
Neutral detergent fiber	48.58 ± 0.18	45.14 ± 0.15	48.77 ± 0.12	52.28 ± 0.09
Acid detergent fiber	31.16 ± 0.16	28.66 ± 0.20	31.58 ± 0.11	34.22 ± 0.14
Hemicellulose	17.42 ± 0.10	16.48 ± 0.11	17.20 ± 0.15	18.06 ± 0.06
Cellulose	19.44 ± 0.15	18.11 ± 0.12	20.22 ± 0.10	21.81 ± 0.11
Total ash	12.25 ± 0.08	12.01 ± 0.12	12.21 ± 0.11	12.44 ± 0.09

**Table 3:** Nutrient intake and digestibility in different groups: GI (control), GII (60R:40C with 10% of concentrate replaced with ML), GIII (70R:30C and 20% replacement of concentrate with ML), and GIV (80R:20C and 20% replacement of concentrate with ML).

Parameters	GI	GII	GIII	GIV	SEM	*p*-Value
Intake (g/d)						
Dry matter	694.67 ^b^ ± 5.43	753.14 ^a^ ± 4.25	752.65 ^a^ ± 9.55	722.88 ^b^ ± 3.77	5.97	0.01
Organic matter	613.65 ± 6.19	663.18 ± 6.28	660.95 ± 21.56	633.63 ± 4.81	7.09	0.07
Crude protein	110.39 ^b^ ± 3.09	123.51 ^a^ ± 1.17	118.86 ^a^ ± 3.88	109.88 ^b^ ± 0.83	1.78	<0.001
Neutral detergent fiber	339.39 ^b^ ± 6.51	339.92 ^a^ ± 3.22	366.96 ^a^ ± 11.97	377.98 ^b^ ± 2.87	5.31	0.01
Acid detergent fiber	216.85 ^b^ ± 6.07	215.11 ^a^ ± 2.04	236.62 ^a^ ± 7.72	246.40 ^b^ ± 1.87	3.83	<0.001
Total digestible nutrients	420.08 ^c^ ± 3.19	472.25 ^a^ ± 13.84	452.27 ^ab^ ± 14.75	422.98 ^bc^ ± 11.85	6.53	0.01
Digestibility (%)						
Dry matter	66.84 ^c^ ± 0.44	69.70 ^ab^ ± 1.10	70.82 ^a^ ± 1.26	67.68 ^bc^ ± 0.38	0.55	0.02
Organic matter	69.56 ^b^ ± 0.68	71.89 ^a^ ± 0.29	71.76 ^a^ ± 0.61	69.75 ^b^ ± 0.62	0.36	0.01
Crude protein	66.79 ± 1.01	65.47 ± 0.64	65.08 ± 0.59	66.19 ± 1.09	0.42	0.52
Ether extract	73.28 ± 0.65	74.72 ± 2.27	75.18 ± 0.69	73.86 ± 2.18	0.77	0.84
Neutral detergent fiber	52.94 ^b^ ± 0.45	54.91 ^a^ ± 0.90	56.04 ^a^ ± 0.77	53.35 ^b^ ± 0.70	0.44	0.02
Acid detergent fiber	40.26 ± 1.87	42.88 ± 2.48	43.63 ± 2.16	41.79 ± 1.60	1.50	0.68

Means bearing different superscripts a, b, and c in the same row differ significantly (*p* < 0.05).

**Table 4:** Growth performance of goats during the feed trial: GI (control), GII (60R:40C with 10% of concentrate replaced with ML), GIII (70R:30C and 20% replacement of concentrate with ML), and GIV (80R:20C and 20% replacement of concentrate with ML).

Attributes	GI	GII	GIII	GIV	SEM	*p*-Value
Initial BW (kg)	14.13 ± 0.90	14.32 ± 0.61	14.36 ± 0.38	14.56 ± 0.89	0.61	0.981
Final BW (kg)	28.43 ^b^ ± 0.89	32.50 ^a^ ± 0.98	31.84 ^a^ ± 0.54	29.15 ^b^ ± 0.84	0.72	0.005
Overall weight gain (kg)	14.13 ^b^ ± 0.46	18.18 ^a^ ± 0.57	17.48 ^a^ ± 0.85	14.59 ^b^ ± 0.89	0.56	0.004
Average daily gain (g)	79.22 ^b^ ± 1.13	101.01 ^a^ ± 3.02	95.68 ^a^ ± 2.16	81.42 ^b^ ± 1.28	1.87	<0.001
Feed conversion efficiency	11.38 ^b^ ± 1.02	13.41 ^a^ ± 0.98	13.15 ^a^ ± 0.01	11.21 ^b^ ± 0.03	0.45	<0.003

Means bearing different superscripts a and b in the same row differ significantly (*p* < 0.05).

**Table 5:** N balance of experimental animals in different groups fed with replacement of concentrate with *Moringa oleifera* leaves: GI (control), GII (60R:40C with 10% of concentrate replaced with ML), GIII (70R:30C and 20% replacement of concentrate with ML), and GIV (80R:20C and 20% replacement of concentrate with ML).

Parameter	GI	GII	GIII	GIV	SEM	*p*-Value
N intake (g/d)	17.75 ^b^ ± 0.45	19.87 ^a^ ± 0.20	19.13 ^a^ ± 0.59	17.67 ^b^ ± 0.14	0.28	<0.001
Fecal N (g/d)	5.42 ± 0.21	5.56 ± 0.12	5.32 ± 0.12	4.96 ± 0.09	0.08	0.06
Urinary (g/d)	10.04 ± 0.66	11.44 ± 0.33	11.21 ± 0.64	10.27 ± 0.16	0.27	0.17
Total N loss (g/d)	15.46 ^b^ ± 0.47	17.01 ^a^ ± 0.26	16.53 ^a^ ± 0.62	15.23 ^b^ ± 0.12	0.25	0.02
N balance (g/d)	2.29 ^b^ ± 0.15	2.87 ^a^ ± 0.10	2.60 ^ab^ ± 0.05	2.44 ^b^ ± 0.13	0.07	0.01
Absorbed N (%)	69.28 ± 1.87	72.01 ± 0.75	72.09 ± 1.04	71.91 ± 0.67	0.61	0.30
Nitrogen retention						
Intake (%)	12.94 ± 0.88	14.47 ± 0.59	13.69 ± 0.62	13.78 ± 0.61	0.34	0.49
Absorbed (%)	18.81 ± 0.57	20.12 ± 0.93	19.04 ± 1.09	19.15 ± 0.76	0.53	0.85

Means bearing different superscripts a and b in the same row differ significantly (*p* < 0.05).

**Table 6:** Enteric methane emissions of male kids in different groups fed with replacement of concentrate with *Moringa oleifera* leaves.

Parameter	GI	GII	GIII	GIV	SEM	*p*-Value
CH_4_(g/d)	8.83 ± 0.07	8.24 ± 0.32	8.13 ± 0.22	8.77 ± 0.13	0.17	0.09
CH_4_ (MJ/d)	0.49 ± 0.03	0.45 ± 0.02	0.45 ± 0.01	0.48 ± 0.01	0.02	0.12
CH_4_ (g/kg DMI)	12.28 ± 0.62	11.42 ± 0.65	11.34 ± 0.73	11.50 ± 0.06	2.08	0.06
CH_4_/Kg (DM digested)	17.34 ± 0.84	15.01 ± 0.83	15.09 ± 0.36	16.67 ± 0.32	0.57	0.08
CH_4_ (g/Kg OMI)	13.85 ± 0.35	12.21 ± 0.49	12.14 ± 0.79	13.84 ± 0.14	0.44	0.06
CH_4_ (g/kg NDFI)	25.31 ± 0.65	24.16 ± 0.90	22.21 ± 0.87	23.30 ± 0.33	0.67	0.08
CH4 energy loss as %						
GE loss	2.68 ± 0.31	2.57 ± 0.10	2.55 ± 0.07	2.90 ± 0.03	0.12	0.471
DE loss	4.73 ± 0.26	4.38 ± 0.14	4.39 ± 0.06	4.70 ± 0.09	0.13	0.296
ME loss	5.91 ± 0.12	5.44 ± 0.24	5.29 ± 0.17	5.83 ± 0.01	0.13	0.07

**Table 7:** Effects of replacement of concentrate with *Moringa oleifera* leaves on blood biochemical parameters, enzymes, and antioxidant activity of goat kids.

Attribute	GI	GII	GIII	GIV	SEM	T	P	T*P
Glucose (mg/dL)	64.56 ± 0.35	64.54 ± 0.30	65.01 ± 0.34	64.76 ± 0.31	0.32	0.327	0.351	0.275
Total protein (g/dL)	6.74 ^b^ ± 0.02	6.97 ^a^ ± 0.04	6.99 ^a^ ± 0.05	6.77 ^b^ ± 0.02	0.03	<0.001	<0.001	<0.001
Plasma albumin (g/dL)	3.38 ^b^ ± 0.03	3.66 ^a^ ± 0.06	3.66 ^a^ ± 0.05	3.44 ^b^ ± 0.03	0.02	0.001	<0.012	<0.001
Plasma globulin (g/dL)	3.36 ± 0.03	3.31 ± 0.03	3.32 ± 0.03	3.34 ± 0.02	0.02	0.232	<0.001	0.120
Albumin globulin ratio	1.01 ± 0.2	1.12 ± 0.03	1.12 ± 0.02	1.03 ± 0.01	0.06	<0.001	<0.145	0.225
Plasma cholesterol (g/dL)	97.36 ^a^ ± 0.25	93.70 ^b^ ± 0.42	92.36 ^c^ ± 0.53	93.89 ^b^ ± 0.32	0.34	<0.001	<0.001	<0.001
Blood plasma AST (U/L)	195.29 ± 1.04	194.17 ± 0.93	194.49 ± 0.89	194.790.89	0.36	0.680	<0.001	0.478
Plasma ALT (U/L)	30.10 ± 0.35	29.92 ± 0.32	30.08 ± 0.27	29.99 ± 0.29	0.29	0.910	<0.001	0.239
Blood plasma ALP (U/L)	101.33 ± 0.56	100.75 ± 0.50	100.49 ± 0.54	100.85 ± 0.56	0.52	0.479	<0.001	0.698
GPx activity (μmol of NDDPH oxidized/gHb)	14.17 ^b^ ± 0.18	14.80 ^a^ ± 0.25	15.03 ^a^ ± 0.27	14.68 ^a^ ± 0.23	0.21	<0.001	<0.001	0.090
Catalase activity (µmol of H2O2 consumed/min/g/Hb)	54.17 ^b^ ± 0.18	54.80 ^a^ ± 0.25	55.03 ^a^ ± 0.27	54.68 ^a^ ± 0.23	0.22	<0.001	<0.001	0.085
SOD activity (U/mg Hb)	91.69 ^b^ ± 0.29	93.28 ^a^ ± 0.30	93.40 ^a^ ± 0.34	93.04 ^a^ ± 0.31	0.29	0.003	<0.001	0.025

**Disclaimer/Publisher’s Note:** The statements, opinions and data contained in all publications are solely those of the individual author(s) and contributor(s) and not of MDPI and/or the editor(s). MDPI and/or the editor(s) disclaim responsibility for any injury to people or property resulting from any ideas, methods, instructions or products referred to in the content.

## Data Availability

The data related to this study are included in this article and the Supplementary Materials.

## Supplementary Materials

The following supporting information can be downloaded at: https://www.mdpi.com/article/10.3390/ani13010097/s1, Table S1: Proximate mineral composition of berseem fodder, concentrate and *Moringa* leaves; Table S2: Chemical composition (%) of berseem fodder, Concentrate and *Moringa* leaves; Table S3: Effect of replacement of concentrate with *Moringa oleifera* leaves on cell mediated immunity of goat kids; GI (control), GII (60R:40C with 10% of concentrate was replaced by ML, GIII (70R:30C and 20% replacement of concentrate with ML), GIV (80R:20C and 20% concentrate mixture was replaced with ML); Table S4: Effect of replacement of concentrate with *Moringa oleifera* leaves on humoral immunity of goat kids; GI (control), GII (60R:40C with 10% of concentrate was replaced by ML, GIII (70R:30C and 20% replacement of concentrate with ML), GIV (80R:20C and 20% concentrate mixture was replaced with ML).

## References

[1] Ouchene-Khelifi N.A., Ouchene N., Lafri M. (2021). Characterization and typology of goat production systems in Algeria based on producers survey. Bull. Natl. Res. Cent..

[2] Makkar H.P.S. (2018). Review: Feed demand landscape and implications of food-not feed strategy for food security and climate change. Animal.

[3] Beauchet-Filleau A.H., Rinne M., Lamminen M., Mapato C., Ampapon T., Wanapat M., Vanhatalo A. (2018). Review: Alternative and novel feeds for ruminants: Nutritive value, product quality and environmental aspects. Animal.

[4] Totakul P., Viennasay B., Sommai S., Matra M., Infascelli F., Wanapat M. (2020). Chaya (*Cnidoscolus aconitifolius*, Mill. Johnston) pellet supplementation improved rumen fermentation, milk yield and milk composition of lactating dairy cows. Livestock Sci..

[5] Huang H., Szumacher-Strabel M., Patra A.K., Ślusarczyk S., Lechniak D., Vazirigohar M., Varadyova Z., Kozłowska M., Cieślak A. (2021). Chemical and phytochemical composition, in vitro ruminal fermentation, methane production, and nutrient degradability of fresh and ensiled *Paulownia* hybrid leaves. Anim. Feed. Sci. Technol..

[6] Puchalska J., Szumacher-Strabel M., Patra A.K., Ślusarczyk S., Gao M., Petrič D., Nabzdyk M., Cieślak A. (2021). The Effect of Different Concentrations of Total Polyphenols from *Paulownia* Hybrid Leaves on Ruminal Fermentation, Methane Production and Microorganisms. Animals.

[7] Abbas R.K., Elsharbasy F.S., Fadlelmula A.A. (2008). Nutritional values of *Moringa oleifera*, total protein. Amino acid, vitamins, minerals, carbohydrates, total fat and crude fiber, under the semi-arid conditions of Sudan. J. Microb. Biochem. Technol..

[8] Padayachee B., Baijnath H. (2020). An updated comprehensive review of the medicinal, phytochemical and pharmacological properties of *Moringa oleifera*. S. Afr. J. Bot..

[9] Olusanya R.N., Kolanisi U., Van Onselen A., Ngobese N.Z., Siwela M. (2020). Nutritional composition and consumer acceptability of *Moringa oleifera* leaf powder (MOLP)-supplemented mahewu. S. Afr. J. Bot..

[10] Moyo B., Oyedemi D., Masika P.J., Muchenje V. (2012). Polyphenolic content and antioxidant properties of *Moringa oleifera* leaf extracts and enzymatic activity of liver from goats supplemented with *Moringa oleifera* leaves/sunflower seed cake. Meat Sci..

[11] Nouman W., Basra S.M.A., Siddiqui M.T., Yasmeen A., Gull T., Alcayde M.A.C. (2014). Potential of *Moringa oleifera* L. as livestock fodder crop: A review. Turk. J. Agric. For..

[12] Kekana T.W., Marume U., Muya M.C., Nherera-Chokuda F.V. (2020). Periparturient antioxidant enzymes, haematological profile and milk production of dairy cows supplemented with *Moringa oleifera* leaf meal. Anim. Feed. Sci. Technol..

[13] Al-Juhaimi F.Y., Alsawmahi O.N., Abdoun K.A., Ghafoor K., Babiker E.E. (2020). Antioxidant potential of moringa leaves for improvement of milk and serum quality of aardi goats. S. Afr. J Bot..

[14] Kholif A.E., Gouda G.A., Olafadehan O.A., Abdo M.M. (2018). Effects of replacement of *Moringa oleifera* for berseem clover in the diets of Nubian goats on feed utilisation, and milk yield, composition and fatty acid profile. Animal.

[15] Min B.R., Solaiman S., Waldrip H.M., Parker D., Todd R.W., Brauer D. (2020). Dietary mitigation of enteric methane emissions from ruminants: A review of plant tannin mitigation options. Anim. Nut..

[16] Knapp J.R., Laur G.L., Vadas P.A., Weiss W.P., Tricarico J.M. (2014). Invited review: Enteric methane in dairy cattle production: Quantifying the opportunities and impact of reducing emissions. J. Dairy Sci..

[17] Ku-Vera J.C., Jiménez-Ocampo R., Valencia-Salazar S.S., Montoya-Flores M.D., Molina-Botero I.C., Arango J., Gómez-Bravo C.A., Aguilar-Pérez C.F., Solorio-Sánchez F.J. (2020). Role of secondary plant metabolites on enteric methane mitigation in ruminants. Front. Vet. Sci..

[18] Abdel-Raheem S.M., Hassan E.H. (2021). Effects of dietary inclusion of *Moringa oleifera* leaf meal on nutrient digestibility, rumen fermentation, ruminal enzyme activities and growth performance of buffalo calves. Saudi J. Biol. Sci..

[19] Kholif A.E., Gouda G.A., Morsy T.A., Salem A.Z.M., Lopez S., Kholif A.M. (2015). *Moringa oleifera* leaf meal as a protein source in lactating goat’s diets: Feed intake, digestibility, ruminal fermentation, milk yield and composition, and its fatty acids profile. Small Rum. Res..

[20] Cohen-Zinder M., Leibovich H., Vaknin Y., Sagi G., Shabtay A., Ben-Meir Y., Nikbachat M., Portnik Y., Yishay M., Miron J. (2016). Effect of feeding lactating cows with ensiled mixture of *Moringa oleifera*, wheat hay and molasses, on digestibility and efficiency of milk production. Anim. Feed. Sci. Technol..

[21] Dong L., Zhang T., Diao Q. (2019). Effect of Dietary Supplementation of *Moringa Oleifera* on the Production Performance and Fecal Methanogenic Community of Lactating Dairy Cows. Animals.

[22] Zeng B., Luo J., Wang P., Yang L., Chen T., Sun J. (2019). The beneficial effects of *Moringa oleifera* leaf on reproductive performance in mice. Food Sci. Nutr..

[23] Afzal A., Hussain T., Hameed A., Shahzad M., Mazhar M.U., Yang G. (2022). Dietary *Moringa oleifera* alters periparturient plasma and milk biochemical indicators and promotes productive performance in goats. Front. Vet. Sci..

[24] Nwinuka N.M., Ibeh G.O., Ekeke G.I. (2005). Proximate composition and levels of some toxicants in four commonly consumed spices. J. Appl. Sci. Env. Manag..

[25] Baccou J.C., Lambert F., Sauvaire Y. (1977). Spectrophotometric method for the determination of total steroidal sapogenin. Analyst.

[26] Folin O., Ciocalteu V. (1927). On tyrosine and tryptophane determinations in proteins. J. Biol. Chem..

[27] Zhishen J., Mengcheng T., Jianming W. (1999). The determination of flavonoid contents in mulberry and their scavenging effects on superoxide radicals. Food Chem..

[28] Sultana S. (2020). Nutritional and functional properties of *Moringa oleifera*. Metab. Open.

[29] Association of Official Analytical Chemists (2005). Official Methods of Analysis.

[30] Van Soest P.J., Robertson J.B., Lewis B.A. (1991). Methods for dietary fiber, neutral detergent fiber, and nonstarch polysaccharides in relation to animal nutrition. J. Dairy Sci..

[31] Johnson K., Huyler M., Westberg H., Lamb B., Zimmerman P. (1994). Measurement of methane emissions from ruminant livestock using a sulfur hexafluoride tracer technique. Environ. Sci. Technol..

[32] Johnson K., Westberg H., Michal J., Cossalman M., Makkar H.P., Vercoe P.E. (2007). The SF6 tracer technique: Methane measurement from ruminants. Measuring Methane Production from Ruminants.

[33] Paglia D.E., Valentine W.N. (1967). Studies on the quantitative and qualitative characterization of erythrocyte glutathione peroxidase. J. Lab. Clin. Med..

[34] Aebi H. (1984). Catalase in vitro. Methods Enzymol..

[35] Madesh M., Balasubramanian K.A. (1998). Microtiter plate assay for superoxide dismutase using MTT reduction by superoxide. Indian J. Biochem. Biophys..

[36] Pattanaik A.K., Khan S.A., Goswami T.K. (2011). Iodine supplementation to a diet containing *Leucaena leucocephala* leaf meal: Consequences on nutrient metabolism, clinical chemistry and immunity of goats. Anim. Prod. Sci..

[37] Sharma A.N., Kumari L.V., Ram C., Mondal G. (2020). Response of different synbiotics on gut health, immunity and growth performance of pre-ruminant Buffalo calves. Indian J. Anim. Nutr..

[38] Abou-Elkhair R., Abdo Basha H., Slouma Hamouda Abd El Naby W., Ajarem J.S., Maodaa S.N., Allam A.A., Naiel M.A.E. (2020). Effect of a diet supplemented with the *Moringa oleifera* seed powder on the performance, egg quality, and gene expression in Japanese laying quail under heat-stress. Animals.

[39] Van Emon M., Sanford C., McCoski S. (2020). Impacts of bovine trace mineral supplementation on maternal and offspring production and health. Animals.

[40] Kholif A.E., Gouda G.A., Galyean M.L., Anele U.Y., Morsy T.A. (2019). Extract of *Moringa oleifera* leaves increases milk production and enhances milk fatty acid profile of Nubian goats. Agrofor. Syst..

[41] Bodas R., Prieto N., García-González R., Andrés S., Giráldez F.J., López S. (2012). Manipulation of rumen fermentation and methane production with plant secondary metabolites. Anim. Feed. Sci. Technol..

[42] Sultana N., Alimon A., Huque K., Sazili A., Yaakub H., Hossain J., Baba M. (2015). The feeding value of Moringa (*Moringa oleifera*) foliage as replacement to conventional concentrate diet in Bengal goats. Adv. Anim. Vet. Sci..

[43] Shankhpal S.S., Waghela C.R., Sherasia P.L., Sridhar V., Srivastava A.K., Singh D. (2019). Effect of feeding Moringa (*Moringa oleifera*) as green fodder on feed intake, milk yield, microbial protein synthesis and blood profile in crossbred cows. Indian J. Anim. Nut..

[44] Kholif A.E., Morsy T.A., Gouda G.A., Anele U.Y., Galyean M.L. (2016). Effect of feeding diets with processed *Moringa oleifera* meal as protein source in lactating Anglo-Nubian goats. Anim. Feed Sci. Technol..

[45] Rahayu I., Timotius K.H. (2022). Phytochemical Analysis, Antimutagenic and antiviral activity of *Moringa oleifera* L. leaf infusion: In Vitro and in silico studies. Molecules.

[46] Fadiyimu A.A., Alokan J.A., Fajemisin A.N. (2010). Digestibility, nitrogen balance and haematological profile of West African dwarf sheep fed dietary levels of *Moringa oleifera* as supplement to *Panicum maximum*. J. Am. Sci..

[47] Hassan H.M., El-Moniary M.M., Hamouda Y., El-Daly E.F., Youssef A.W., Abd El-Azeem N.A. (2016). Effect of different levels of *Moringa oleifera* leaves meal on productive performance, carcass characteristics and some blood parameters of broiler chicks reared under heat stress conditions. Asian J. Anim. Vet. Adv..

[48] Aregheore E.M. (2020). Intake and digestibility of *Moringa oleifera*–batiki grass mixtures by growing goats. Small Rumin. Res..

[49] Elghandour M.M.Y., Vallejo L.H., Salem A.Z.M., Mellado M., Camacho L.M., Cipriano M., Olafadehan O.A., Olivares J., Rojas S. (2017). *Moringa oleifera* leaf meal as an environmental friendly protein source for ruminants: Biomethane and carbon dioxide production, and fermentation characteristics. J. Clean. Prod..

[50] Zeng B., Sun J.J., Chen T., Sun B.L., He Q., Chen X.Y. (2018). Effects of *M. oleifera* silage on milk yield, nutrient digestibility and serum biochemical indexes of lactating dairy cows. J. Anim. Physiol. Anim. Nut..

[51] Kholif A.E., Gouda G.A., Abu Elella A.A., Patra A.K. (2022). Replacing the Concentrate Feed Mixture with *Moringa oleifera* Leaves Silage and *Chlorella vulgaris* Microalgae Mixture in Diets of Damascus Goats: Lactation Performance, Nutrient Utilization, and Ruminal Fermentation. Animals.

[52] Machmüller A., Ossowski D.A., Kreuzer M. (2000). Comparative evaluation of the effects of coconut oil, oilseeds and crystalline fat on methane release, digestion and energy balance in lambs. Anim. Feed. Sci. Technol..

[53] Goel G., Makkar H.P.S. (2012). Methane mitigation from ruminants using tannins and saponins. Trop. Anim. Health Prod..

[54] Pedraza-Hernández J., Elghandour M.M.M.Y., Khusro A., Camacho-Diaz L.M., Vallejo L.H., Barbabosa-Pliego A. (2019). Mitigation of ruminal biogases production from goats using *Moringa oleifera* extract and live yeast culture for a cleaner agriculture environment. J. Clean. Prod..

[55] Ndlovu T., Chimonyo M., Okoh A.I., Muchenje V., Dzama K., Dube S. (2009). A comparison of nutritionally related blood metabolites among Nguni, Bonsmara and Angus steers raised on sweetveld. Vet. J..

[56] Bashar M.K., Huque K.S., Sarker N.R., Sultana N. (2020). Quality assessment and feeding impact of Moringa feed on intake, digestibility, enteric CH_4_ emission, rumen fermentation, and milk yield. J. Adv. Vet. Anim. Res..

[57] Babiker E.E., Juhaimi F.A.L., Ghafoor K., Mohamed H.E., Abdoun K.A. (2016). Effect of partial replacement of alfalfa hay with *Moringa* species leaves on milk yield and composition of Najdi ewes. Trop. Anim. Health Prod..

[58] Giannini E.G., Testa R., Savarino V. (2005). Liver enzyme alteration: A guide for clinicians. CMAJ.

[59] Melesse A., Getye Y., Berihun K., Banerjee S. (2013). Effect of feeding graded levels of *Moringa stenopetala* leaf meal on growth performance, carcass traits and some serum biochemical parameters of Koekoek chickens. Livestock Sci..

[60] Sun B., Zhang Y., Ding M., Xi Q., Liu G., Li Y. (2018). Effects of *Moringa oleifera* leaves as a substitute for alfalfa meal on nutrient digestibility, growth performance, carcass trait, meat quality, antioxidant capacity and biochemical parameters of rabbits. J. Anim. Physiol. Anim. Nut..

[61] Osman H.M., Shayoub M.E., Babiker E.M. (2012). The effect of *Moringa oleifera* leaves on blood parameters and body weights of Albino rats and rabbits. Jordan J. Biol. Sci..

[62] Moyo B., Masika P.J., Muchenje V. (2012). Effect of supplementing crossbred Xhosa lop-eared goat castrates with *Moringa oleifera* leaves on growth performance, carcass and non-carcass characteristics. Trop. Anim. Health Prod..

[63] Afzal A., Hussain T., Hameed A. (2021). *Moringa oleifera* supplementation improves antioxidant status and biochemical indices by attenuating early pregnancy stress in beetal goats. Front. Nut..

[64] Vergara-Jimenez M., Almatrafi M.M., Fernandez M.L. (2017). Bioactive components in *Moringa Oleifera* leaves protect against chronic disease. Antioxidants.

