# Supplementary material for: Impact of Moringa oleifera Leaves on Nutrient Utilization, Enteric Methane Emissions, and Performance of Goat Kids
Source: Animals (Basel). 2022 Dec 27;13(1):97. doi: 10.3390/ani13010097 (PMC9817938; doi:10.3390/ani13010097)
Supplement: Supplementary file 1 [file animals-13-00097-s001.zip › animals-1832678-supplementary.pdf]

Table S1: Proximate mineral composition of berseem fodder, concentrate and *Moringa leaves*

Parameter	Concentrate mixture	Berseem fodder	*Moringa oleifera*
Calcium (%)	1.37±0.05	1.92±0.02	1.85±0.12
Phosphorus (%)	0.79±0.10	0.38±0.04	0.15±0.05
Magnesium (%)	0.81±0.03	0.40±0.03	4.81±0.14
Iron (ppm)	287±0.50	289.53±0.65	330±0.33
Copper (ppm)	29.21±0.23	4.61±0.06	9.21±0.23
Zinc (ppm)	49±0.45	16.41±0.23	26.72±0.31

Table S2: Chemical composition (%) of berseem fodder, Concentrate and *Moringa leaves.*

Attribute	Berseem	Concentrate feed	*Moringa oleifera*
Dry matter (%)	23.61±0.12	91.21±0.18	29.26±0.20
Organic matter (%)	87.22±0.07	89.26±0.10	89.62±0.15
Crude protein (%)	17.56±0.12	20.14±0.08	23.37±0.13
Ethyl ether (%)	2.98 ±0.07	4.45±0.09	7.13±0.12
Neutral detergent fiber (%)	59.29±0.17	23.58±0.21	26.86±0.20
Acid detergent fiber (%)	39.33±0.17	11.69±0.14	18.70±0.25
Total ash (%)	12.78±0.07	10.74±0.08	10.03±0.06

Table S3. Effect of replacement of concentrate with *Moringa oleifera* leaves on cell mediated immunity of goat kids; G1 (control), GII (60R:40C with 10% of concentrate was replaced by ML, GIII (70R:30C and 20% replacement of concentrate with ML), GIV (80R:20C and 20% concentrate mixture was replaced with ML).

Absolute reading (mm) hour after phytohemagglutinin-P injection							
Attribute	G I	G II	GIII	G IV	Treat	Period	T*P
0 hour	5.79±0.29	5.60±0.28	5.73±0.32	5.99±0.31			
6 hours	7.01±0.06	7.48±0.52	8.13±0.35	7.67±0.52			
12 hours	7.83±0.12	8.55±0.42	9.03±0.36	8.78±0.50			
24 hours	7.98^{b}±0.29	8.8^{ab}±0.34	9.50^{a}±0.45	8.59^{ab}±0.14	<0.001	<0.001	0.831
48 hours	7.30^{b}±0.28	7.96^{ab}±0.22	8.70^{a}±0.41	7.84^{ab}±0.18			
72 hours	6.59±0.13	7.01±0.28	7.41±0.34	6.85±0.30			
Mean±SE	7.08^{c}±0.15	7.57^{b}±0.23	8.08^{a}±0.25	7.62^{b}±0.21			

Means with different superscripts a and b in the same row differ significantly (p<0.05)

Table S4: Effect of replacement of concentrate with *Moringa oleifera* leaves on humoral immunity of goat kids; G1 (control), GII (60R:40C with 10% of concentrate was replaced by ML, GIII (70R:30C and 20% replacement of concentrate with ML), GIV (80R:20C and 20% concentrate mixture was replaced with ML)

Attribute	G I	GII	GIII	G IV	Treat	Period	T*P
Hemagglutination (HA) titre, log $_2$ against sheep-Red Blood Cells							
7 days	1.04±0.13	1.10±0.13	1.07±0.11	1.22±0.09			
14 days	1.73±0.06	1.91±0.09	2.20±0.08	1.95±0.05	<0.001	<0.001	0.11
21 days	1.14±0.10	1.44±0.10	1.57±0.09	1.26±0.08			
Mean±SE	1.30^{b}±0.09	1.48^{a}±0.10	1.61^{a}±0.12	1.48^{a}±0.09			

Means with different superscripts a and b in the same row differ significantly (p<0.05)
